# Accuracy of manual and automatic placement of an anatomical coordinate system for the full or partial radius in 3D space

**DOI:** 10.1038/s41598-020-65060-7

**Published:** 2020-05-15

**Authors:** Marieke G. A. de Roo, Johannes G. G. Dobbe, Abbas Peymani, Anne D. van der Made, Simon D. Strackee, Geert J. Streekstra

**Affiliations:** 1 Amsterdam UMC, University of Amsterdam, Department of Plastic, Reconstructive and Hand Surgery, Amsterdam Movement Sciences, Meibergdreef 9, Amsterdam, the Netherlands; 2 Amsterdam UMC, University of Amsterdam, Department of Biomedical Engineering and Physics, Amsterdam Movement Sciences, Meibergdreef 9, Amsterdam, the Netherlands; 3 Amsterdam UMC, University of Amsterdam, Department of Orthopaedic Surgery, Amsterdam Movement Sciences, Meibergdreef 9, Amsterdam, the Netherlands

**Keywords:** Bone, Skeleton, Trauma

## Abstract

Accurate placement of a coordinate system on the radius is important to quantitatively report 3D surgical planning parameters or joint kinematics using 4D imaging techniques. In clinical practice, the scanned length of the radial shaft varies among scanning protocols and scientific studies. The error in positioning a radial coordinate system using a partially scanned radius is unknown. This study investigates whether the imaged length of the radius significantly affects the positioning of the coordinate system. For different lengths of the radius, the error of positioning a coordinate system was determined when placed automatically or manually. A total of 85 healthy radii were systematically shortened until 10% of the distal radius remained. Coordinate systems were placed automatically and manually at each shortening step. A linear mixed model was used to associate the positioning error with the length of the radial shaft. The accuracy and precision of radial coordinate system placement were compared between automatic and manual placement. For automatic placement of the radial coordinate system, an increasing positioning error was associated with an increased shortening of the radius (P = < 0.001). Automatic placement is superior to manual placement; however, if less than 20% of the radial shaft length remains, manual placement is more accurate.

## Introduction

The orientation of the radial coordinate system (CS) is first described by Kobayashi *et al*.^1^ and is used in a wide array of research fields, from planning corrective osteotomies of the radius^2–5^ to describing carpal kinematics^1,6–11^. Variable placement methods of a CS, e.g. manual selection of anatomical landmarks^9,12–15^, marker based^16,17^, or algorithm based^3,5,18,19^, could hamper the comparison of positioning parameters among published studies and among patients within a study. Research in which knee kinematics was observed, demonstrated that uniform positioning of a CS is important since changes in coordinate system placement result in rotation differences of up to 27° for the same motion in the same patient^20^. The International Society of Biomechanics proposed joint CS guidelines for the radius in an effort to facilitate a better comparison of research results^21^. These guidelines are based on a completely scanned radius. As Coburn *et al*.^22^ previously stated, the scanned length of the radial shaft varies among scanning protocols and among patient groups in scientific studies^4^. Therefore, if only a partially scanned radius is available for research purposes, it is important to know the additional error when describing repositioning parameters in the case of corrective osteotomies or when carpal kinematics are described. The main purpose of this study was to quantify the radial coordinate system (RCS) positioning error that is expected to occur with a shortening of the radial shaft for both manual-, and automatic RCS placement. We hypothesized that the length of the scanned radial shaft significantly affects the accuracy and precision of positioning an RCS. To that end, we investigated 1) if automatic RCS placement is affected by the available length of the radial shaft, 2) if the accuracy of placing an RCS is different for manual placement and automatic placement, and 3) if automatic RCS placement errors depend on gender, age and the presence of a growth plate.

## Materials and methods

To investigate the accuracy of placing an RCS, we first obtained a virtual model of a radius by CT scanning of the arm and performing subsequent image segmentation. The 3D radius model was shortened by clipping. Automatic placement of the RCS was based on the inertial axes of the (shortened) radius model, while manual placement was done selecting osseous landmarks in virtual space with a computer program. All of the above steps and requirements are detailed below.

### Data acquisition and segmentation

The contralateral CT scans of anonymised patients (N = 85) (42 male (29 right, 13 left), 43 female (24 right, 19 left), mean age of 27 years (standard deviation: 15 years, range: 7 – 72 years)) who were previously treated at our institute between March 2012 and June 2017 for reconstructive surgery of a malunited radius were used in this study. In 33 patients a growth plate was present. According to the Dutch Medical Research Involving Human Subjects Act, no approval of the medical ethics committee was required. A radius was eligible for inclusion when it was completely scanned and there was no history of trauma, fracture, or known growth defects. All left radii were mirrored to facilitate identical data analysis for left and right radii. A regular dose, high-resolution computed tomographic (CT) scan (Philips Brilliance 64 CT scanner, Cleveland, OH; voxel size 0.45 ×0.45 ×0.45 mm^3^, 120 kV, 150 mAs, pitch 0.6) was used to scan patients in prone position with the arm extended above the head. A semi-automatic method was used to segment each radius. First, a threshold-connected region growing algorithm was started. A binary closing algorithm was used to fill residual holes and close the outline, which was followed by a Laplacian level-set segmentation growth algorithm, according to the method described by Dobbe JG, *et al*.^2^ Segmentation produces a hollow virtual model of the bone, with vertices at the outline of the mesh. This results in a virtual radius model of the cortex for further data analysis in 3D. All image analyses described in this article were performed with dedicated custom-made software^2^.

### Automatic RCS positioning & clipping

**Figure 1:**
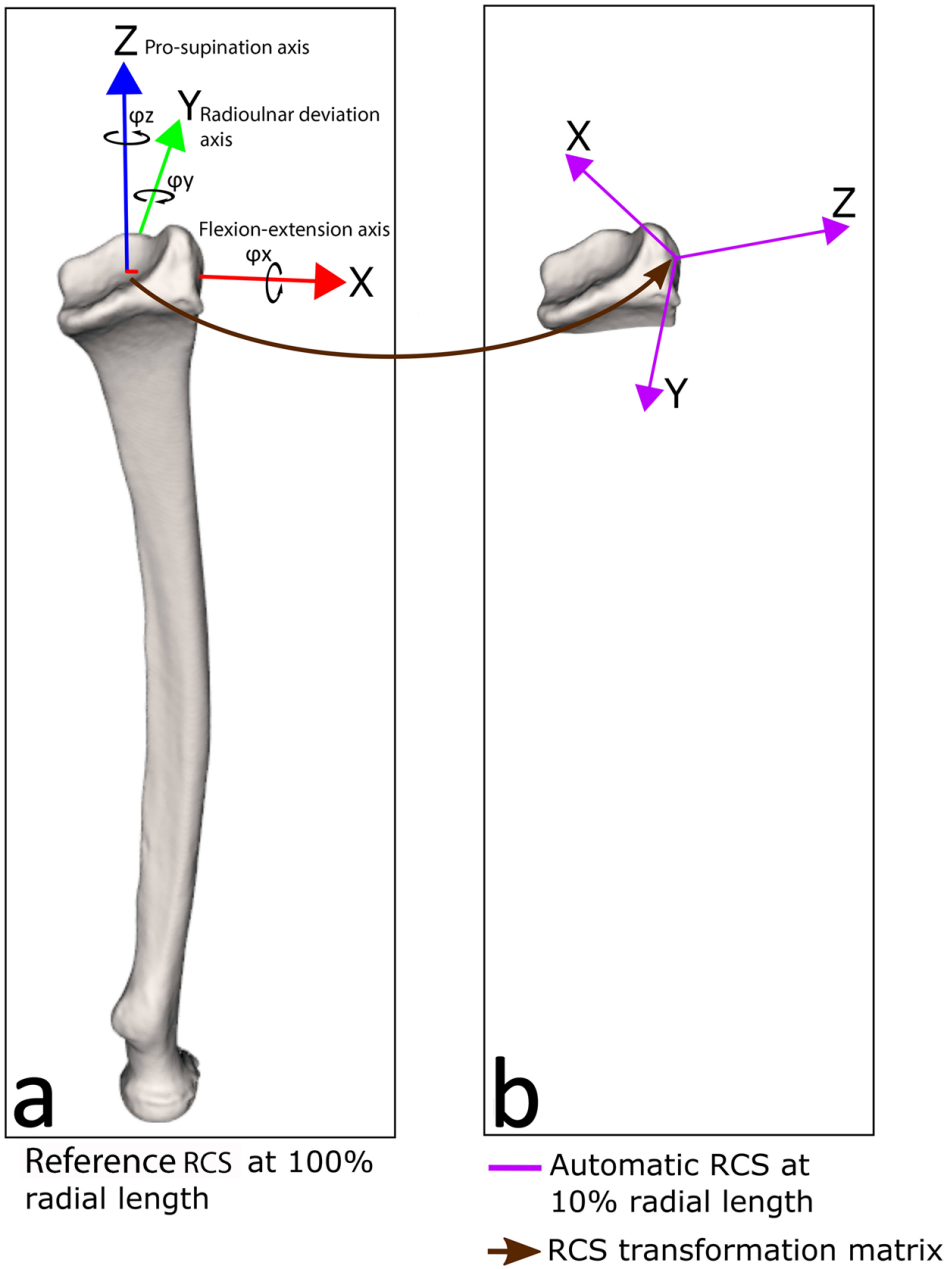
(**a**) Anatomical coordinate system of the radius based on the entire radius. The longitudinal inertial axis of the radius is the z-axis (pronation (z-) – supination (z+) axis). The x-axis (flexion (x+)- extension (x-) axis) is directed towards the highest point of the radius model, the radial styloid. The y-axis (radial (y+)- ulnar (y-) deviation axis) is the axis perpendicular to the z- and x-axes. Rotations around the axes are defined as ϕx, ϕy, and ϕz (black arrows). Translations along these axes are defined as Δx, Δy and Δz. (**b**) Extreme example of an automatic RCS positioning error when the radial length is only 10% of the total length. The left coordinate system (multicolour) is the reference standard. The right coordinate system (purple) represents the coordinate system placed by the algorithm on a shortened radius. The brown arrow represents the transformation matrix, which yields the translation error (d_err_) and the rotation error (φ_err_).

After segmentation, a coordinate system was placed automatically using the anatomical features of the 3D radius model. This automatic algorithm starts by calculating the inertia tensor using the points in the segmented 3D radius model. This tensor enables the calculation of the three eigenvectors and eigenvalues. The vector with the smallest eigenvalue points in the direction of the bone axis and represents the z-axis of the initial RCS. The remaining two eigenvectors and the centroid define the temporary x- and y-axes, and the origin of the RCS. A CS-to-CS transformation matrix is subsequently performed to align the radius model and temporary RCS with the global CT coordinate system. The z-axis of the temporary RCS is subsequently translated to the centroid of the distal 15% of the available radius object, having a length of at least 20 mm (or whatever is available if the total length drops below this limit). This requires knowing the length of the radius, which was calculated from the points in the transformed radius mesh with the highest and lowest z-coordinate. The above procedure caused the z-axis of the temporary RCS to intersect the transformed distal radius between the two fossae. The origin of this temporary RCS was then placed at this intersection point. The algorithm then rotated the x-axis about the z-axis to the styloid, which was identified as the point in the radius model with the highest z-coordinate. The x-axis was pointed towards the styloid but stayed perpendicular to the z-axis. The y-axis is perpendicular to the x- and z-axes following the right-hand rule. This temporary RCS if finally transformed back to the actual bone using the CS-to-CS transformation matrix and defines the actual RCS. Eigenvector analysis, as used in the above procedure, provides the orientation of the RCS z-axis, but not the +z or –z direction. In this study all patients are scanned in prone position with the arm extended above the head. The current software therefore chooses the +z axis of the RCS as the direction that best agrees with the +z axis of the CT coordinate system. The software allows adapting the axes directions in case a patient is scanned in an alternative way. The x-, y- and z-axes are defined in agreement with the axes of flexion-extension-, radioulnar deviation- and pro- supination, respectively. The RCS placed by the algorithm on a complete radius served as the reference CS in this study (Fig. 1).

**Figure 2:**
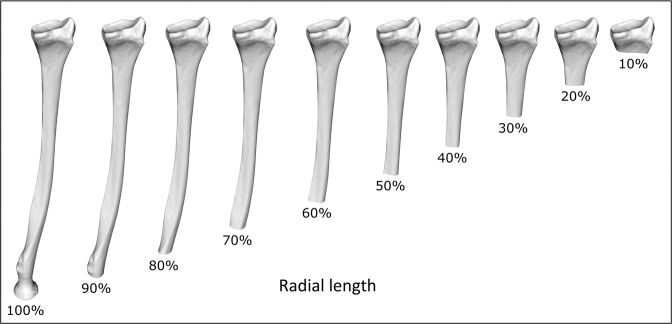
Systematic shortening of the radial shaft by a digital cutting algorithm. The radius is shortened in steps of 10% by removing the proximal part of the radius model.

The radii were subsequently shortened in 10% steps by clipping the proximal end from the 3D radius model (Fig. 2). The RCS was then determined again, either automatically or manually, using the (shortened) radius models.

### Effect of segmentation on RCS placement

One radius of a cadaver arm was scanned 10 times. The scans were obtained with the same scanner and scanning protocol used for obtaining patient data. All 10 scans were segmented, and the bone model was subsequently shortened in steps of 10% using the methods described above. Then, the algorithm was used to position RCSs for each radial length, and the positioning variability was calculated.

### Automatic and manual RCS placement

For automatic RCS placement, coordinate systems were placed on 85 healthy radii. The algorithm calculated the RCS for each radius model, resulting in 10 RCSs for each radius. Three physicians of the hand surgery department and orthopaedic surgery department manually positioned RCSs on 5 radii that were randomly selected out of the available 85 radii. Each observer repeated this positioning 10 times (for each radial length). This resulted in 150 sets of coordinates (3 observers analysing 5 radii 10 times each). One observer repeated all positioning steps 3 times, with a minimal time interval of one month, to calculate intra-observer variability. The 5 radii were randomly presented at 10% radial length. The distal segment of the radius was presented in 3D space without a coordinate system present. With a mouse click on a button in the software user interface, a coordinate system was placed randomly in 3D space. The observer was then able to set a cursor in 3D space at an osseous landmark and move the origin of the CS to that location. The observer was further able to orient each axis interactively. When reorienting an axis, the observer was asked whether that axis was allowed to reorient freely in 3D space or to stay in a plane perpendicular to one of the other two axes, which effectively kept the non-reoriented axes fixed in 3D space. The observer was instructed to place the RCS according to the ISB recommendations^21^ using the osseous landmarks of the radius: the origin of the RCS is located on the ridge between the radioscaphoid fossa and the radiolunate fossa, with the z-axis pointing in the distal direction. The x-axis is oriented towards the tip of the radial styloid^12^. The y-axis positioned perpendicular to the x- and z- axes.

Subsequently, the five radii at 20% radial length were shown randomly. The steps were repeated and the lengths of the radii increased until the final placement steps, when the full lengths of the radii were shown to the observers. No identification number was present on the 3D radial models to minimize recall bias. All three observers work daily with three-dimensional bone models in imaging software. A training environment was created in the software to practice positioning the RCS at multiple levels of the radial length; these training sets were not included in our evaluation dataset. Each observer was given unlimited time to familiarize themselves with the software and tools for placing the coordinate system. A handout was made available with instructions for RCS positioning. It included a graphical example of RCS positioning for a radius that was not included in their dataset.

### Quantification of the RCS positioning error

The positioning errors were determined by calculating the CS100-to-CSx transformation matrix, where CS100 is the coordinate system for the entire radius (reference standard) and CSx is the coordinate system for the radius segment at relative length x (10%-90%) (Fig. 1). This matrix yielded the translation errors, (Δx, Δy, Δz) and rotation errors (Δϕx, Δϕy, Δϕz; our visualization toolbox uses the rotation sequence y, x, z). The Euclidian distance served as the total translation error ${d}_{err}=\sqrt{{(\Delta x)}^{2}+{(\Delta y)}^{2}+{(\Delta z)}^{2}}$. The total rotation error was represented by ${\varphi }_{err}=\sqrt{{(\Delta {\varphi }_{x})}^{2}+{(\Delta {\varphi }_{y})}^{2}+{(\Delta {\varphi }_{z})}^{2}}$ as proposed by^23^. From the individual error estimates, the accuracy (median, since we calculated with absolute numbers) and precision (dispersion, presented as interquartile range, with minimal and maximal errors) of RCS positioning were calculated. Subsequently, the effect of gender, age, and growth plate on RCS placement was analysed.

### Statistical analysis

Four statistical models were used to answer the research questions. First, to determine the association of the radial length to the translation errors (Δx, Δy, Δz, d_err_) and the rotation errors (Δϕx, Δϕy, Δϕz, ϕ_err_) of automatic RCS positioning, a linear mixed model analysis was used. The percentage of radial length was included as a fixed factor, the number of radial bones as a random factor, and the translation errors and rotation errors as dependent factors. A likelihood ratio test using ANOVA compared the effect of the fixed factors on the translation and rotation errors^24^. Next, to evaluate the results of manual positioning, the association of the length of the radial shaft on the translation errors and the rotation errors was assessed with linear mixed model analysis. The length of the radius was used as a fixed factor, the number of radial bones as a random factor, and the translation errors and rotation errors as dependent factors. A likelihood ratio test using ANOVA tested the association. Finally, to compare RCS positioning accuracy, significant differences in the translation and rotation errors of manual positioning in comparison to automatic positioning were calculated with a Mann-Whitney U test. The precision of RCS positioning was evaluated by comparing the dispersions of d_err_ and ϕ_err_ between manual and automatic positioning using the Ansari and Bradley test^25^. Non-parametric tests were applied for the comparison between automatic and manual RCS positioning, due to the difference in sample size (85 versus 5 radii). To calculate the intra-observer and inter-observer variability for RCS positioning, the interclass correlation coefficient was calculated for the total translation and total rotation error in RCS positioning (ICC, two-way mixed, random effects model, absolute agreement), for a full-length radius and the overall error in a shortened radius (for all radial lengths). The ICC can be interpreted according to the method of Landis and Koch, as it is the parametric analogue of the chance-corrected kappa measure of agreement^26^, as follows: poor (0 to 0.20), fair (0.21-0.40), moderate (0.41 to 0.60), good (0.61 to 0.80) and perfect (0.81 to 1) agreement.

Again, the associations of gender, age, and the presence of a growth plate on the rotation and translation errors were determined with linear mixed model analysis. Gender, age, and the presence of a growth plate were alternately included as fixed factors, the number of radial bones as a random factor, and the d_err_ and ϕ_err_ as dependent factors. Again, the likelihood ratio test with ANOVA was used to test the associations. Statistical analysis was performed with R version 3.3.

## Results

### Effect of segmentation on RCS placement

**Figure 3:**
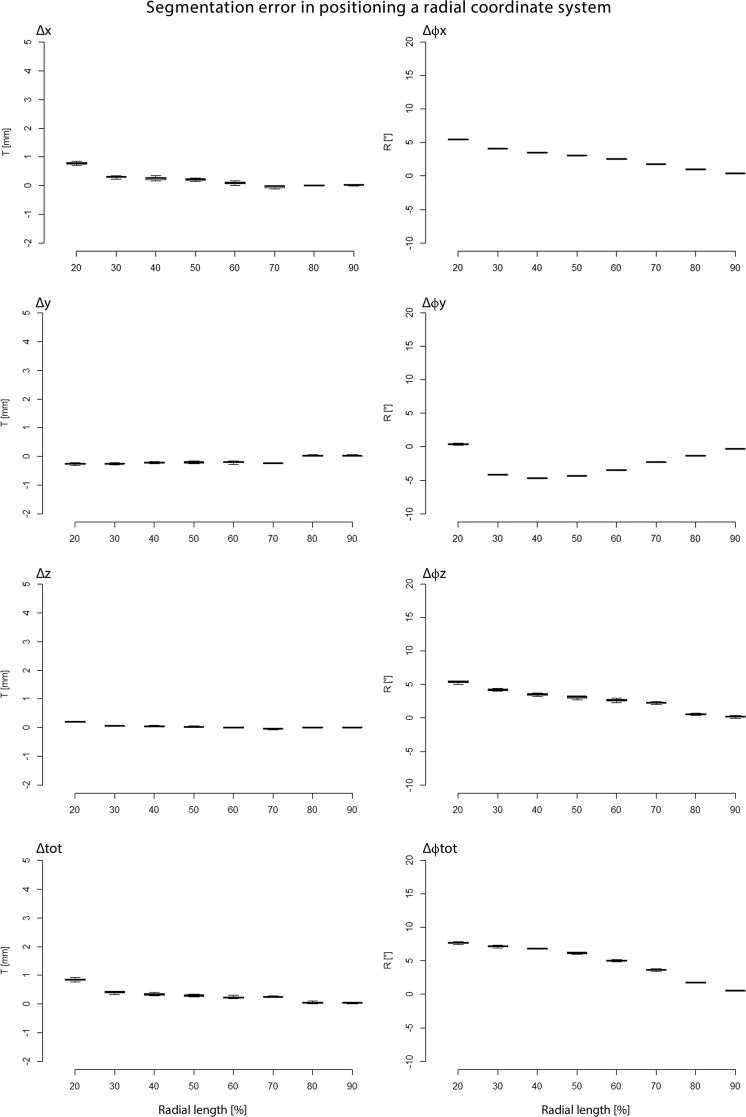
Error in automatic RCS placement after repeated segmentation. The x-axis represents the radial length in percentages. The y-axis represents the rotation or translation error in degrees or millimetres, respectively. Δx, Δϕx = translation-, rotation along the flexion-extension-axis; Δy, Δϕy, = translation-, rotation along the radioulnar deviation axis; Δz, Δϕz = translation-, rotation along the pro-supination axis; and Δtot = total translation error; Δϕtot = total rotation error.

**Figure 4:**
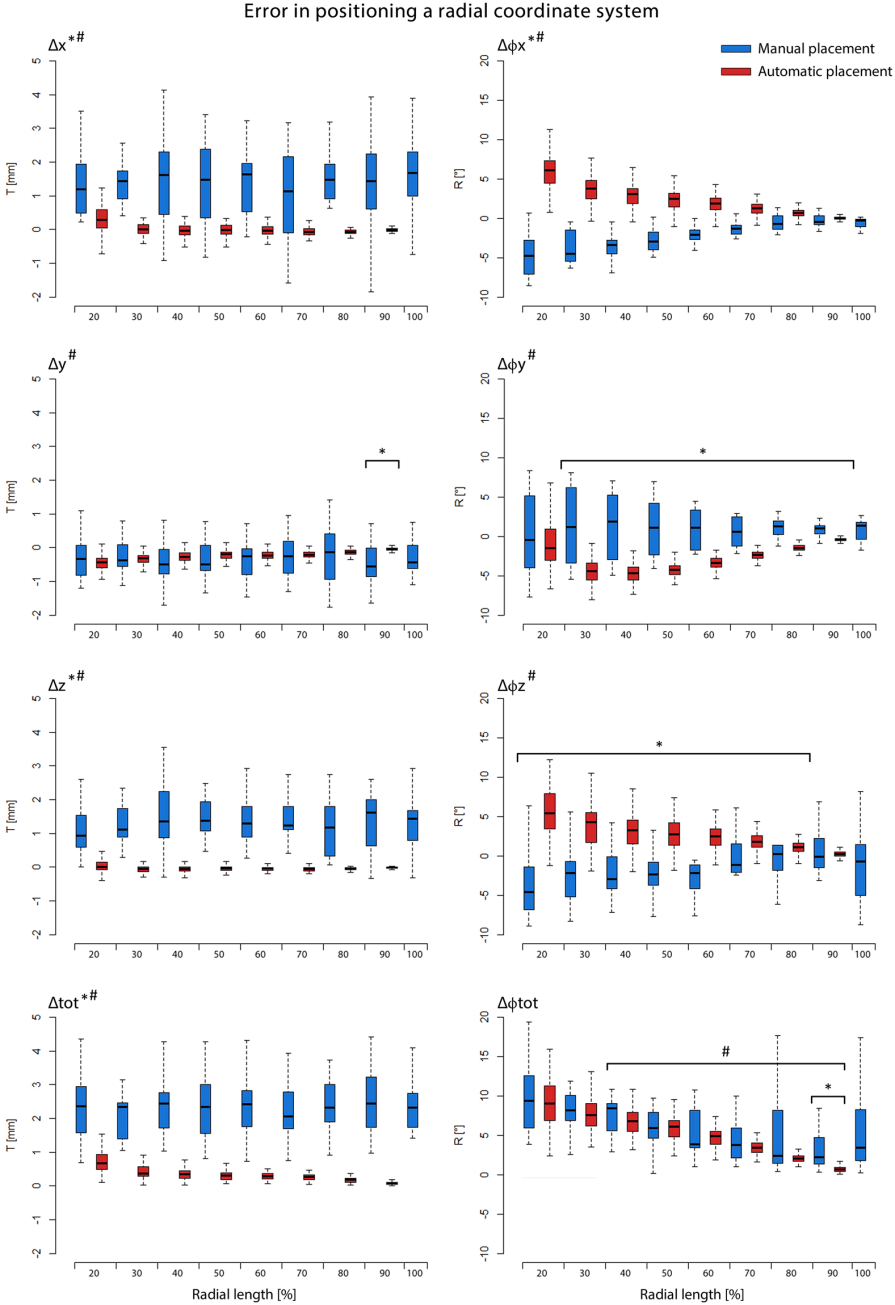
Error in positioning a radial coordinate system automatically (red boxplots) or manually (blue boxplots) represented by translation errors in the left column and by rotation errors in the right column. Δx, Δϕx = translation-, rotation along the flexion-extension-axis; Δy, Δϕy, = translation-, rotation along the radioulnar deviation axis; Δz, Δϕz = translation-, rotation along the pro-supination axis; and Δtot = total translation error; Δϕtot = total rotation error. Significant differences (p < 0.05) in the accuracy (median) of RCS positioning are indicated by a star (*). Significant differences (p < 0.05) in the precision (dispersion) of RCS positioning are indicated by a hash (#).

Figure 3 shows the error in automatic RCS placement after repeated segmentation (n = 10). This variability appears to be small compared to the variability due to manual or automatic RCS placement (Fig. 4). This renders the method sufficiently robust for our evaluation experiments.

### Automatic RCS placement

The patient characteristic data were normally distributed. Shortening of the radius was associated with an increase of Δx, Δy, Δz, and d_err_ (p =< 0.001) and of Δϕx, Δϕy, Δϕz, and ϕ_err_ (p =< 0.001) (Fig. 4). A relatively large error in RCS positioning was present when 10% of the radial shaft remained, with Δx: 15.2 mm; Δy: 1.9 mm; Δz: 1.5 mm; d_err_ of 15.9 mm (SD 1,6 mm) and Δϕx: 20.6°; Δϕy: 74.6°; Δϕz: 168.4° and a ϕ_err_ of 190° (SD 14°) in comparison to the reference standard (Fig. 1b). All results are detailed in supplemental table 1. Age, gender, and the presence of a growth plate did not influence the translation or rotation errors.

### Manual RCS placement

Shortening of the radial shaft was associated with an increasing RCS rotation error (Δϕx, Δϕz, ϕ_err_, p=<0.05, Δϕy, p = 0.4), but not associated with an increasing RCS translation error (p=>0.5). The average total translation and rotation error for all lengths of the shortened radii was approximately 2.4 mm (SD 1 mm) and 6.8° (SD 4.7°). When 100% of the radius was present, the total translation- and rotation error was 2.5 mm (SD 1 mm) and 5.3° (SD 4.7°). All results are detailed in supplemental table 1. When the accuracy of RCS positioning was compared between manual and automatic positioning, manual RCS positioning was only more accurate when <20% of the distal radial length remained (d_err_, p=<0.001 and ϕ_err_, p=<0.001) (Fig. 3). If the translation and rotation errors are evaluated separately, automatic positioning was significantly more accurate in minimizing the translation error (d_err_) when 20% to 90% of the radial length was available (p < 0.001). However, there was no significant difference in the accuracy of the total rotation error (ϕ_err_) if 20% to 80% of the radial shaft was present. When the precision of RCS placement was analysed, there was a significant difference (p < 0.05) in the dispersion of the rotation error between 90% to 40% radial length in favour of automatic positioning. With less than <20% radial length, manual positioning outperformed automatic positioning. The dispersion of the translation errors for all radial lengths was significantly different (p < 0.05), with 90% to 20% radial length in favour of automatic positioning and <20% radial length in favour of manual positioning. The overall inter-observer ICC for the total translation error (d_err_) for all lengths of the radius was 0.504, and total rotation error (ϕ_err_) 0.540, both with moderate agreement. The inter-observer ICC when 100% of the radius was presented was 0.775 for total translation error (d_err_) and 0.674 for total rotation error (ϕ_err_), both with good agreement. The overall intra-observer ICC for total translation error (d_err_) for all lengths of the radius was 0.691, with good agreement; and total rotation error (ϕ_err_) 0.537, with moderate agreement. The intra-observer ICC when 100% of the radius was presented was 0.901 for the total translation error (d_err_), with perfect agreement; and 0.640 for total rotation error (ϕ_err_), with good agreement.

## Discussion

The aim of this study was to analyse the effect of radial length on the positioning error of an anatomical coordinate system for the radius. We found that manual placement of the RCS was better only if less than 20% of the radial length was available. In all other cases, automatic placement of the RCS was either the same or better in terms of accuracy and/or precision. We therefore recommend using automatic RCS placement for future research unless the available radial length is less than 20%. We further found that gender, age or the presence of a growth plate does not influence automatic RCS placement. Whether the RCS translation error is relevant depends on the application. If the RCS is used to report the *absolute* position of, e.g., carpal bones in 4D imaging, and the RCS is manually placed, this results in approximately a 2.4 mm error when reporting carpal positions. Likewise, if the research evaluates *absolute* position changes, e.g., in comparing radius malunion reconstruction parameters, the RCS translation will have an effect on the reported parameters^27^. However, if the RCS is used to quantify the degree of malunion by reporting the *relative* translation of the distal bone with respect to the proximal bone segment, the RCS translation has no effect on the reported relative parameters and is therefore irrelevant^10,28^. On the other hand, the rotation error of the RCS always affects the positioning parameters irrespective of whether they represent absolute or relative positioning. Therefore, the choice to make a partial scan of the radius in an attempt to reduce the radiation dose may result in a poor definition of the RCS and hence a misinterpretation of the reconstruction parameters. The large RCS positioning error that occurs with automatic placement when less than 20% of the radial length is available is inherent to the implementation of our algorithm for automatic RCS placement since detection of the bone axis based on the inertia tensor is no longer accurate if a small bone segment remains. The algorithm further translates the z-axis to the centroid of the distal 15% of the radius with a length of at least 20 mm. If the length of this segment is compromised, as is the case if 10% of the radius remains, the algorithm uses the points available, resulting in an additional RCS translation error. When 100% of the radius was presented, the RCS positioning inter- and intra-observer agreement was good. Unfortunately, comparable research on inter- and intra-observer agreement on manual CS positioning on the radius is lacking. Our findings are comparable to those of the patella^29^, femur^30^ and tibia^30,31^, with mean inter-observer variabilities ranging from 1.2 to 3.5 mm translation errors of osseous landmarks. We did not find research in which the observer agreement was determined for manual CS positioning on a partial bone. In this study, only 3 physicians positioned the coordinate system on 5 shortened radii, which can be considered a limitation. However, the large difference in the translation errors found between automatic positioning and manual positioning is evident. Shortening of the radial shaft had a significant effect on accurate RCS positioning, with automatic RCS placement being superior to manual placement. However, the automatic RCS positioning error becomes substantial if <20% of the distal radial shaft is available, in which case the RCS should be placed manually.

## Supplementary information

Supplementary Table 1.
