# Supplementary material for: Accuracy of manual and automatic placement of an anatomical coordinate system for the full or partial radius in 3D space
Source: Sci Rep. 2020 May 15;10:8114. doi: 10.1038/s41598-020-65060-7 (PMC7229017; doi:10.1038/s41598-020-65060-7)
Supplement: Supplementary file 1 — Supplementary Table 1. [file 41598_2020_65060_MOESM1_ESM.docx]

**Title:** Accuracy of manual and automatic placement of an anatomical coordinate system for the full or partial radius in 3D space

**Author list**: Marieke G.A. de Roo*^1,2^, Johannes G.G. Dobbe^2^, Abbas Peymani^1,2^, Anne D. van der Made^3^, Simon D. Strackee^1^, Geert J. Streekstra^2^

**Author affiliations:**^1^ Amsterdam UMC, University of Amsterdam, Department of Plastic, Reconstructive and Hand Surgery, Amsterdam Movement Sciences, Meibergdreef 9, Amsterdam, the Netherlands

^2^ Amsterdam UMC, University of Amsterdam, Department of Biomedical Engineering and Physics, Amsterdam Movement Sciences, Meibergdreef 9, Amsterdam, the Netherlands

^3^ Amsterdam UMC, University of Amsterdam, Department of Orthopaedic Surgery, Amsterdam Movement Sciences, Meibergdreef 9, Amsterdam, the Netherlands

| **Supplemental table 1.** Influence of length of the radius on coordinate system placement | | | | | |
| --- | --- | --- | --- | --- | --- |
| Radial length | Positioning error | PC | Human | Mann Withney-U  p-value | Ansari Bradley  P-value |
| 100 | ΔX | Gold standard | 1.7 [1.0; 2.3] |  |  |
|  | ΔY | Gold standard | -0.4 [ -0.6; 0.08] |  |  |
|  | ΔZ | Gold standard | 1.4 [0.8; 1.7] |  |  |
|  | ΔϕX | Gold standard | -0.3 [-1.0; -0.1] |  |  |
|  | ΔϕY | Gold standard | 1.3 [-0.3; 1.8] |  |  |
|  | ΔϕZ | Gold standard | -0.7 [-5.0; 1.4] |  |  |
|  | Total translation | Gold standard | 2.3 [1.7; 2.8] |  |  |
|  | Total rotation | Gold standard | 3.4 [1.8; 8.3] |  |  |
| 90 | ΔX | - 0.02 [- 0.05; 0.03] | 1.4 [0.6; 2.3] | <0.05 | <0.05 |
|  | ΔY | - 0.04 [- 0.07; -0.01] | - 0.5 [- 0.9; - 0.004] | <0.05 | <0.05 |
|  | ΔZ | - 0.01 [- 0.03; 0.001] | 1.6 [0.6; 2.0] | <0.05 | <0.05 |
|  | ΔϕX | 0.07 [-0.07; 0.2] | -0.4 [-0.8; 0.3] | <0.05 | <0.05 |
|  | ΔϕY | -0.4 [-0.5; -0.3] | 1.1 [0.3; 1.4] | <0.05 | <0.05 |
|  | ΔϕZ | 0.2 [-0.01; 0.5] | -0.1 [-1.5; 2.2] | 0.66 | <0.05 |
|  | Total translation | 0.08 [0.05; 0.1] | 2.4 [1.7; 3.2] | <0.05 | <0.05 |
|  | Total rotation | 0.6 [0.4; 0.9] | 2.2 [1.4; 4.7] | <0.05 | <0.05 |
| 80 | ΔX | -0.07 [-0.1; -0.01] | 1.5 [0.9; 2.0] | <0.05 | <0.05 |
|  | ΔY | -0.1 [-0.2; -0.08] | -0.1 [-0.9; 0.4] | 0.9 | <0.05 |
|  | ΔZ | -0.05 [-0.08; -0.02] | 1.2 [0.3; 1.8] | <0.05 | <0.05 |
|  | ΔϕX | 0.7 [0.3; 1.0] | -0.7 [-1.4; 0.3] | <0.05 | <0.05 |
|  | ΔϕY | -1.5 [-1.7; -1.2] | 1.3 [0.2; 1.9] | <0.05 | <0.05 |
|  | ΔϕZ | 1.1 [0.5; 1.6] | 0.2 [-1.8; 1.3] | <0.05 | <0.05 |
|  | Total translation | 0.2 [0.1;0.2] | 2.3 [1.9; 3.0] | <0.05 | <0.05 |
|  | Total rotation | 2.1 [1.7; 2.4] | 2.4 [1.4; 8.1] | 0.26 | <0.05 |
| 70 | ΔX | -0.07 [-0.2; 0.02] | 1.1 [-0.1; 2.2] | <0.05 | <0.05 |
|  | ΔY | -0.2 [-0.3; -0.1] | -0.3 [-0.8; 0.2] | 0.83 | <0.05 |
|  | ΔZ | -0.06 [-0.1; -0.02] | 1.2 [1.1; 1.8] | <0.05 | <0.05 |
|  | ΔϕX | 1.3 [0.7; 1.8] | -1.3 [-2.0; -0.9] | <0.05 | <0.05 |
|  | ΔϕY | -2.4 [-2.8; -2.0] | 0.6 [-1.2; 2.5] | <0.05 | <0.05 |
|  | ΔϕZ | 1.8 [1.0; 2.5] | -1.1 [-2.1; 1.5] | <0.05 | <0.05 |
|  | Total translation | 0.3 [0.2; 0.3] | 2.1 [1.7; 2.8] | <0.05 | <0.05 |
|  | Total rotation | 3.5 [2.8; 4.0] | 3.8 [2.1; 5.9] | 0.83 | <0.05 |
| 60 | ΔX | -0.03 [-0.1; 0.1] | 1.6 [0.5; 2.0] | <0.05 | <0.05 |
|  | ΔY | -0.2 [-0.3; -0.1] | -0.2 [-0.8; -0.03] | 0.7 | <0.05 |
|  | ΔZ | -0.04 [-0.1; -0.003] | 1.3 [0.9; 1.8] | <0.05 | <0.05 |
|  | ΔϕX | 1.9 [1.1; 2.6] | -2.1 [-2.7; -1.5] | <0.05 | <0.05 |
|  | ΔϕY | -3.3 [-3.9; -2.8] | 1.1 [-1.7; 3.3] | <0.05 | <0.05 |
|  | ΔϕZ | 2.4 [1.3; 3.4] | -2.1 [-4.1; -1.1] | <0.05 | <0.05 |
|  | Total translation | 0.3 [0.2; 0.4] | 2.4 [1.8; 2.8] | <0.05 | <0.05 |
|  | Total rotation | 4.9 [3.8; 5.5] | 3.9 [3.5; 8.2] | 0.49 | <0.05 |
| 50 | ΔX | -0.01 [-0.1; 0.1] | 1.5 [0.3; 2.4] | <0.05 | <0.05 |
|  | ΔY | -0.2 [-0.3; -0.1] | -0.5 [-0.6; 0.1] | 0.1 | <0.05 |
|  | ΔZ | -0.04 [-0.1; 0.01] | 1.4 [1.1; 1.9] | <0.05 | <0.05 |
|  | ΔϕX | 2.5 [1.5; 3.2] | -2.9 [-3.9; -1.8] | <0.05 | <0.05 |
|  | ΔϕY | -4.3 [-4.8; -3.7] | 1.1 [-2.3; 4.2] | <0.05 | <0.05 |
|  | ΔϕZ | 2.8 [1.4; 4.2] | -2.4 [-3.8; -0.8] | <0.05 | <0.05 |
|  | Total translation | 0.3 [0.2; 0.4] | 2.3 [1.6; 3.0] | <0.05 | <0.05 |
|  | Total rotation | 6.1 [4.8; 6.9] | 6.0 [4.6; 7.9] | 0.23 | <0.05 |
| 40 | ΔX | -0.03 [-0.2; 0.1] | 1.6 [0.5; 2.3] | <0.05 | <0.05 |
|  | ΔY | -0.3 [-0.4; -0.2] | -0.5 [-0.8; -0.06] | 0.07 | <0.05 |
|  | ΔZ | -0.04 [-0.1; 0.0] | 1.4 [0.9;2.2] | <0.05 | <0.05 |
|  | ΔϕX | 3.1 [1.9; 3.8] | -3.4 [-4.5;-2.8] | <0.05 | <0.05 |
|  | ΔϕY | -4.7 [-5.6; -3.9] | 1.9 [-2.9; 5.2] | <0.05 | <0.05 |
|  | ΔϕZ | 3.2 [1.6; 4.6] | -2.9 [-4.2; -0.1] | <0.05 | <0.05 |
|  | Total translation | 0.3 [0.2; 0.5] | 2.4 [1.7; 2.8] | <0.05 | <0.05 |
|  | Total rotation | 6.8 [5.5; 7.9] | 8.5 [5.6; 9.1] | 0.23 | <0.05 |
| 30 | ΔX | 0.002 [-0.1; 0.15] | 1.4 [0.9; 1.7] | <0.05 | <0.05 |
|  | ΔY | -0.4 [-0.6;-0.2] | -0.4 [-0.6; 0.1] | 0.90 | <0.05 |
|  | ΔZ | -0.05 [-0.1; 0.01] | 1.1 [0.9; 1.7] | <0.05 | <0.05 |
|  | ΔϕX | 3.9 [2.5; 4.8] | -4.5 [-5.5; -1.5] | <0.05 | <0.05 |
|  | ΔϕY | -4.4 [-5.5; -3.4] | 1.2 [-3.3; 6.1] | <0.05 | <0.05 |
|  | ΔϕZ | 4.3 [1.7; 5.5] | -2.2 [-5.2; -0.7] | <0.05 | <0.05 |
|  | Total translation | 0.4 [0.3; 0.6] | 2.3 [1.4; 2.5] | <0.05 | <0.05 |
|  | Total rotation | 7.6 [6.2; 9.0] | 8.2 [6.9; 10.1] | 0.57 | 0.41 |
| 20 | ΔX | 0.3 [ 0.1; 0.6] | 1.2 [0.5; 1.9] | <0.05 | <0.05 |
|  | ΔY | -0.4 [-0.6; -0.3] | -0.3 [-0.8; 0.07] | 0.56 | <0.05 |
|  | ΔZ | 0.01 [-0.1; 0.2] | 0.9 [0.6; 1.5] | <0.05 | <0.05 |
|  | ΔϕX | 6.1 [4.5; 7.3] | -4.8 [-7.1; -2.8] | <0.05 | <0.05 |
|  | ΔϕY | -1.5 [-3.1; 0.9] | -0.5 [-4.0; 5.2] | 0.60 | <0.05 |
|  | ΔϕZ | 5.4 [3.4; 7.9] | -4.6 [-6.8; -1.4] | <0.05 | <0.05 |
|  | Total translation | 0.7 [0.5; 1.9] | 2.4 [1.6; 2.9] | <0.05 | <0.05 |
|  | Total rotation | 9.1 [6.9; 11.3] | 9.4 [6.0; 12.6] | 0.67 | 0.24 |
| 10 | ΔX | 15.2 [11.9; 16.9] | 1.6 [0.8; 2.3] | <0.05 | 0.8 |
|  | ΔY | 1.9 [0.9; 2.8] | -0.5 [-0.9; 0.5] | <0.05 | 0.01 |
|  | ΔZ | -1.5 [-4.1; -0.5] | 1.1 [0.8; 2.1] | <0.05 | <0.05 |
|  | ΔϕX | -20.6 [-26.9; -14.3] | -2.5 [-4.4; 0.7] | <0.05 | 0.3 |
|  | ΔϕY | -74.6 [-77.3; -72.8] | 1.9 [0.9; 4.5] | <0.05 | <0.05 |
|  | ΔϕZ | -168.4 [-172.9; 48.6] | -0.1 [-8.7; 3.9] | <0.05 | <0.05 |
|  | Total translation | 15.9 [14.9; 17.1] | 2.7 [1.7; 3.2] | <0.05 | <0.05 |
|  | Total rotation | 189.7 [185.1; 193.2] | 8.3 [5.3; 17.4] | <0.05 | <0.05 |

Parameters ΔX, ΔY, ΔZ and total translation are in millimeters; and ΔϕX , ΔϕY, ΔϕZ and total rotation in degrees. Between the brackets the 25^th^ and 75^th^ quantile are represented.
